# Genomic Prediction and Genome-Wide Association Studies of Flour Yield and Alveograph Quality Traits Using Advanced Winter Wheat Breeding Material

**DOI:** 10.3390/genes10090669

**Published:** 2019-08-31

**Authors:** Peter S. Kristensen, Just Jensen, Jeppe R. Andersen, Carlos Guzmán, Jihad Orabi, Ahmed Jahoor

**Affiliations:** 1 Nordic Seed A/S, 8300 Odder, Denmark; 2 Department of Molecular Biology and Genetics, Aarhus University, 8830 Tjele, Denmark; 3 Departamento de Genética, Escuela Técnica Superior de Ingeniería Agronómica y de Montes, Edificio Gregor Mendel, Campus de Rabanales, Universidad de Córdoba, CeiA3, 14071 Córdoba, Spain; 4 Department of Plant Breeding, The Swedish University of Agricultural Sciences, 23053 Alnarp, Sweden

**Keywords:** wheat breeding, baking quality, Alveograph, flour yield, genomic selection, GWAS

## Abstract

Use of genetic markers and genomic prediction might improve genetic gain for quality traits in wheat breeding programs. Here, flour yield and Alveograph quality traits were inspected in 635 F_6_ winter wheat breeding lines from two breeding cycles. Genome-wide association studies revealed single nucleotide polymorphisms (SNPs) on chromosome 5D significantly associated with flour yield, Alveograph P (dough tenacity), and Alveograph W (dough strength). Additionally, SNPs on chromosome 1D were associated with Alveograph P and W, SNPs on chromosome 1B were associated with Alveograph P, and SNPs on chromosome 4A were associated with Alveograph L (dough extensibility). Predictive abilities based on genomic best linear unbiased prediction (GBLUP) models ranged from 0.50 for flour yield to 0.79 for Alveograph W based on a leave-one-out cross-validation strategy. Predictive abilities were negatively affected by smaller training set sizes, lower genetic relationship between lines in training and validation sets, and by genotype–environment (G×E) interactions. Bayesian Power Lasso models and genomic feature models resulted in similar or slightly improved predictions compared to GBLUP models. SNPs with the largest effects can be used for screening large numbers of lines in early generations in breeding programs to select lines that potentially have good quality traits. In later generations, genomic predictions might be used for a more accurate selection of high quality wheat lines.

## 1. Introduction

Baking quality of wheat is a complex trait controlled by many genes with minor effects and few genes with larger effects [1,2]. The amount and the composition of gluten proteins have large effects on baking quality of wheat. The major gluten loci are the high molecular weight glutenins (HMWGs) *Glu-A1*, *Glu-B1*, and *Glu-D1* and the low molecular weight glutenins (LMWGs) *Glu-A3*, *Glu-B3*, and *Glu-D3* [3,4]. Milling quality and water absorption are affected by the hardness of the grain. Grain hardness is, to a large extent, controlled by the *Hardness* locus on chromosome 5D, consisting of the genes *Pina-D1*, *Pinb-D1*, and *Gsp-1* [5,6].

Breeding for improved wheat quality is challenging, because phenotyping of most quality traits requires laborious analyses of relatively large amounts of grain using expensive equipment. Baking tests can be used for evaluating the quality of wheat lines by determining bread loaf volume and texture. However, breeding programs typically do not have the resources to perform baking tests with large numbers of lines [7]. Another way of testing baking quality is rheological measurements with, for example, an Alveograph. Here, a piece of dough is inflated with air into a bubble, and dough tenacity (Alveo P), extensibility (Alveo L), and strength (Alveo W) are determined. Alveo W and the ratio of Alveo P/L are correlated with bread loaf volume and can give good indications of the quality of wheat lines [8]. Together with protein content and grain hardness, the Alveograph traits can be used for predicting the potential end-use for particular wheat lines [9]. Previously, quantitative trait loci (QTL) for the Alveograph traits were identified on many chromosomes, e.g., on chromosome 1B and 5D for both Alveo P and W. Additionally, QTL were identified on chromosomes 3A, 3B, and 5B for Alveo W and on chromosomes 2A and 2B for Alveo P [10,11,12,13]. Groos et al. (2004) [10], Tadesse et al. (2015) [14], and Zanetti et al. (2001) [13] identified QTL for Alveo L on chromosomes 2B, 3B, 4A, and 5B. Similarly, QTL for flour yield were identified on most chromosomes. In several studies of spring or winter wheat, QTL for flour yield were identified on chromosomes 1B, 2A, 2B, 3B, 4A, 5A, 5D, or 6A [2,12,15,16,17,18,19,20].

Those in Denmark are allowed to use more nitrogen for the cultivation of wheat for bread production if certain criteria are fulfilled. The cultivar must be on a list that has been approved by The Danish Agricultural Agency. For a cultivar to be approved, it must have a bread volume and a Zeleny sedimentation value of, at minimum, 90% of the average of the cultivars that have already been approved, and it must not have sticky dough. In addition, the grain used for bread production should, as a minimum, have a test weight of 78 kg/hL, a falling number of 275 s, and a protein content of 11.5% [21,22]. Therefore, it is important for breeders to be able to select lines with high quality.

The use of DNA markers to predict quality traits can reduce costs and enable higher selection intensities compared to having to do the phenotypic measurements. Thereby, higher numbers of wheat lines might be tested in breeding programs. Marker assisted selection based on few DNA markers can be effective for traits that are controlled by few QTL with large effects [23]. However, many traits are more complex and are controlled by many QTL with small effects. Furthermore, major QTL might be fixed in breeding programs and can therefore not be used for selection. In such cases, genomic predictions based on a large number of genome-wide markers could be a good approach. A training set of lines that have been both genotyped and phenotyped is needed to train a model for prediction of genomic estimated breeding values (GEBVs) in other genotyped lines (validation or breeding set) [24]. Different types of models can be used for genomic predictions, and their accuracies depend on the genetic architecture of the trait and on the relatedness of lines in the training and validation set [25,26]. In genomic best linear unbiased prediction (GBLUP) models, breeding values are predicted based on a genomic relationship matrix (G-matrix) [27]. In Bayesian models, marker effects can be assigned prior distributions that might fit better than a normal distribution for certain traits or populations [28]. Genomic prediction was first implemented in cattle breeding and is now widely used in animal breeding programs [28]. Many studies have been focused on the effectiveness and the optimal use of genomic predictions in different plant species and for different traits [29,30,31,32]. Generally, heritabilities and predictive abilities are lower for complex traits, such as grain yield, than for more simple traits, such as heading date or resistance against certain diseases, which are controlled by both minor and major genes [33,34,35]. For wheat quality traits, predictive abilities are often moderate to high [25,36,37,38]. However, the use of markers with large effects could likely be done in a better way than in the standard GBLUP or Bayesian models, and the optimal implementation strategy might differ between traits and breeding programs [26,34,39,40]. The size and the composition of the training set is crucial for accurate genomic predictions [29,38,41]. A recent study of quality traits and grain yield using wheat hybrids from each of the German quality classes E, A, B, and C reported that genomic predictions within one quality class worked well when the training set contained individuals from the same class. However, for predictions between quality classes, individuals from each of the classes should be included to obtain predictive abilities as high as for the within-class predictions [42].

The aims of the present study were to identify SNPs affecting the baking quality traits flour yield and Alveographs P, L, and W and to evaluate and compare genomic prediction models in order to facilitate implementation of genomic selection for baking quality traits in breeding programs.

## 2. Materials and Methods

### 2.1. Plant Material

In total, 635 F_6_ winter wheat lines from two breeding cycles in the Danish plant breeding company Nordic Seed A/S (Holeby, Denmark) were used in this study. The 321 lines of the first breeding cycle (set2014) were harvested in 2014, and the 314 lines of the second breeding cycle (set2015) were harvested in 2015. Six out of 96 crossing parents were used for both sets (years), while the remaining 90 crossing parents were used for one of the sets only. Each line was grown in an unreplicated 9.9 m^2^ plot at Lolland, Denmark, following standard Danish agricultural practices. Approximately 180 kg of nitrogen were applied per hectare during the growth season, and no irrigation was used.

### 2.2. Phenotyping

Phenotyping was done at the Wheat Chemistry and Quality Laboratory at International Maize and Wheat Improvement Center (CIMMYT), Mexico. Grain samples were conditioned to 13.5% moisture content and then milled one time using a Brabender Quadrumat Jr. (Brabender GmbH & Co. KG, Duisburg, Germany). Flour yield was measured as the percentage of refined flour obtained from each grain sample after the bran fraction was sieved away through a 75 µm mesh sieve. A Chopin Alveograph (Tripette and Renaud, Villeneuve-la-Garenne, France) was used to obtain the Alveograph traits P, L, and W (Alveo P is dough tenacity, Alveo L is dough extensibility, and Alveo W is dough strength) using a modified version of the American Association of Cereal Chemists (AACC) method 54-30A [8,43]. Flour was mixed with a saltwater solution to form a dough, which was cut into discs. After resting 20 min at 25 °C, the dough discs were inflated with air, thus the dough expanded as a bubble. During inflation of each disc, a curve was recorded of pressure inside the bubble until it burst. Alveo P was the maximum height of the curve, Alveo L was the length of the curve, and Alveo W was the area under the curve. The coefficient of variation was calculated for each trait by dividing the standard deviation of the raw phenotypes with the mean.

### 2.3. Genotyping

DNA extraction was performed with a modified cetyl trimethylammonium bromide (CTAB) method [44] using leaves of three bulked, two-week-old seedlings for each line. Genotyping was done by TraitGenetics (Gatersleben, Germany) with the 15K Illumina Infinium iSelect HD Custom Genotyping BeadChip technology. The 13,006 called SNP markers were edited for minor allele frequency (MAF) > 1% and missing values < 10%, and the remaining 10,802 SNPs were used for the analyses. For each line, at least 90% of the SNPs were successfully genotyped.

### 2.4. Statistical Analysis

Genome-wide associations were studied using single marker regression. The following model was run for each of the 10,802 SNPs:

***y*** = ***Xb*** + ***w_i_**a_i_* + ***Z*_1_*u*** + ***e*** (1)

where ***y*** is a vector of observed phenotypes, ***X*** and ***Z*_1_** are design matrices, ***b*** is a vector of fixed effects (mean and year/set), ***w_i_*** is the vector of genotypes of the *i^th^* SNP coded as 1, 0, −1, *a_i_* is the additive genetic effect of the *i^th^* SNP, ***u*** is a vector of additive genetic effects of the lines (***u*** ~ *N*(0,***G*_1_***σ_g_*^2^), where ***G*_1_** is a G-matrix (genomic relationship matrix) and *σ_g_*^2^ is additive genetic variance), and ***e*** is a vector of random residual effects (***e*** ~ *N*(0,***I**σ**_e_***^2^), where **I** is an identity matrix and *σ**_e_***^2^ is the residual variance). Model effects and variance components were estimated by restricted maximum likelihood using the software package DMU [45].

For each chromosome, a G-matrix was calculated based only on the SNPs mapped to the remaining chromosomes and then used to correct for structure when analyzing the SNPs mapped to the excluded chromosome. This was done so that the SNP effect was not included twice in the model. G-matrices were calculated using the first method proposed by Van Raden [27]:

(2) $$G=\frac{Z_{2}Z_{2}^{\prime }}{2\sum p_{i}\left(1-p_{i}\right)}$$

where *p_i_* is the MAF of *i^th^* marker, ***Z*_2_** = ***M** − **P**, **M*** is a matrix with the marker alleles coded as 1, 0, -1, and ***P*** is a matrix where the *i^th^* column contains the MAF of SNP *i* calculated as 2(*p_i_* – 0.5). Missing genotypes were set to 0 in matrix ***Z_2_***.

Genomic inflation factors, λ_IF_, were calculated for each trait by dividing the observed median value of the chi-squared statistic for the SNPs with the expected median value [46]. The inflation factor λ_IF_ was used to correct the *p*-values for inflation by dividing the chi-squared statistic with λ_IF_ and then re-calculating the *p*-values. The significance threshold was set using a Bonferroni correction: 5% divided by number of SNPs (0.05/10,802 = 4.6 × 10^−6^).

DNA sequences surrounding the significantly associated SNPs were blasted against the annotated reference genome of the bread wheat variety *Chinese Spring*, IWGSC RefSeq v1.0 [47], using the BLAST tool of EnsemblPlants [48].

A Bayesian Power Lasso model where all SNPs were fitted simultaneously was also used for genome-wide association analyses in the Bayz software [49]:

***y*** = ***Xb*** + ***Z*_3_*u*** + ***e*** (3)

where ***y*** is a vector of observed phenotypes, ***b*** is a vector of the mean + year/set effect with design matrix **X**, ***Z*_3_** is a matrix of the alleles of the SNPs coded as 0, 1, 2, ***u*** is a vector of additive genetic SNP effects, and ***e*** is a vector of residual effects. The prior distribution of SNP effects was specified as an exponential power distribution [50]:

(4) $$p\left(u\right)=\overset{m}{\underset{i=1}{\prod }}\frac{1}{2}\lambda_{RP}e^{-\lambda_{RP}{\left|u_{i}\right|}^{\beta }}$$

where *m* is the number of markers, and *β* is shape parameter to control the sparsity, which affects the shrinkage of the SNP effects. Setting *β* to 1 makes the model equivalent to a standard Bayesian Lasso model. If *β* is set to less than 1, the difference between large and small marker effects can be increased [50]. Models with *β* of 0.2, 0.4, 0.8, and 1.0 were run, and the Deviance Information Criterion was used to determine the optimal *β* for each trait [51]. Residual effects were assigned a normal prior distribution. The residual variance, the mean, the year/set effect, and the rate parameter, *λ_RP_,* were assigned flat prior distributions. Model parameters were estimated using Markov Chain Monte Carlo with a length of 100,000 with 30,000 cycles as burn-in. The tool pbayz supplied with Bayz was used to compute posterior means, and the R package CODA was used to check for convergence to the posterior distribution [52].

Genomic predictions based on all 10,802 SNPs were conducted using the Bayesian Power Lasso model (3) and using a GBLUP model:

***y*** = ***Xb*** + ***Z*_4_*u*** + ***e*** (5)

where ***y*** is a vector of observed phenotypes, ***X*** and ***Z*_4_** are design matrices, ***b*** is a vector of fixed effect (mean and year/set), ***u*** is a vector of additive genetic effects (***u*** ~ *N*(0 ***G*_2_** $\sigma_{g}^{2}$), where ***G*_2_** is a G-matrix computed as above (2) using all SNPs, $\sigma_{g}^{2}$ is additive genetic variance), and ***e*** is a vector of random residual effects (***e*** ~ *N*(0,***I***σ**_*e*_**^2^)).

Model effects and variance components for the GBLUP and Bayesian Power Lasso models were estimated by DMU and Bayz packages, respectively. For the GBLUP models, the narrow-sense genomic heritability corresponding to records of single plots was calculated as:

(6) $$h^{2}=\frac{d\left(G_{2}\right)\sigma_{g}^{2}}{d(G_{2})\sigma_{g}^{2}+\sigma_{e}^{2}}$$

where *d(**G_*2*_**)* is the average diagonal element of the G-matrix (calculated using all SNPs), $\sigma_{g}^{2}$ is additive genetic variance, and $\sigma_{e}^{2}$ is residual variance.

The following cross-validations (CVs) were used to study the effectiveness of possible strategies for implementing genomic selection in breeding programs:

Leave-one-out (LOO): The GEBV of each line was predicted from the rest of the lines. The training set used in the LOO strategy was as large as possible (634 lines), and the genetic relationship between lines in the training and validation set was higher compared to the other CV strategies.

Leave-family-out (LFO): The GEBVs of lines in each half-sib family were predicted from lines of the remaining families. The average size of the half-sib families was 46 lines. Using this strategy, the effect of the genetic relationship between the lines in training and validation sets was studied.

Leave-set-out (LSO): The GEBVs of lines in each set were predicted from lines from the other set. The training set sizes were 314 or 321 lines. The LSO CV strategy was used for studying the predictive ability when GEBVs of lines from one breeding cycle were predicted from lines from another breeding cycle.

k-fold: The lines were randomly divided into k folds (2, 5, or 10) of equal size. The GEBVs of lines in each fold were predicted from lines in the other folds. The training set sizes were approximately 318 lines for the 2-fold, 508 lines for the 5-fold, and 572 lines for the 10-fold. Approximately half of the lines in the training sets were from set2014 and half from set2015. The k-fold CV strategy was used for studying the effect of the training set size.

Furthermore, the effect of the training set size was studied by selecting from 10% to 90% of the 635 lines as a training set for genomic predictions using LOO CV. The lines were randomly selected, and selection and predictions were repeated 100 times for each 10% interval.

Correlations between observed phenotypes corrected for fixed effects and GEBVs were calculated to determine predictive abilities of the models and were compared with the maximum correlation (the square root of the narrow-sense genomic heritability). Biases of the genomic predictions were calculated as the deviation from the expectation of the slope (1.0) of the regression line of the corrected phenotypes on the GEBVs.

Genomic feature models with two G-matrices were tested in order to possibly utilize the most significant SNPs better [53,54]. The lines were randomly divided in two folds. The lines of one fold were used for genome-wide association studies (GWAS), and the remaining lines were used for LOO genomic predictions. The SNPs used for computing the two G-matrices were selected for each trait based on their *p*-value in the GWAS. The most significant SNPs were used for one G-matrix, and the remaining SNPs were used for the other. The following thresholds for number of SNPs to include in the group of most significant were tested: 5, 10, 50, 100, 500, 1000, 3000, 5000, 7000, 10,000, and all 10,802 SNPs. The following model was used for the genomic predictions:

***y*** = ***Xb*** + ***Z*_5_*s*** + ***Z*_6_*n*** + ***e*** (7)

where ***y*** is a vector of observed phenotypes, ***X***, ***Z*_5_**, and ***Z*_6_** are design matrices, ***b*** is a vector of fixed effect (mean and year/set), ***s*** and ***n*** are vectors of additive genetic effects (***s*** ~ *N(*0*,**G_s_*** $\sigma_{g_{s}}^{2}$*)* and ***n*** ~ *N(*0*,**G_n_*** $\sigma_{g_{n}}^{2}$*),* where ***G_s_*** and ***G_n_*** are G-matrices computed as above (2) using significant (***G_s_***) or nonsignificant (***G_n_***) SNPs, $\sigma_{g_{s}}^{2}$ and $\sigma_{g_{n}}^{2}$ are additive genetic variances, and ***e*** is a vector of random residual effects (***e*** *~ N(*0*,**I**σ**_e_**^2^*)).

## 3. Results

### 3.1. Phenotyping and Genotyping

**Figure 1:**
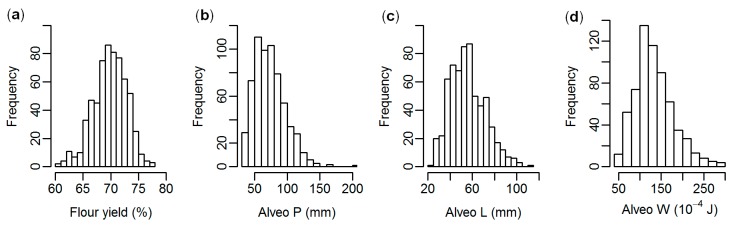
Distribution of phenotypes. (**a**): Flour yield, (**b**): Alveo P (dough tenacity), (**c**): Alveo L (dough extensibility), (**d**): Alveo W (dough strength).

**Figure 2:**
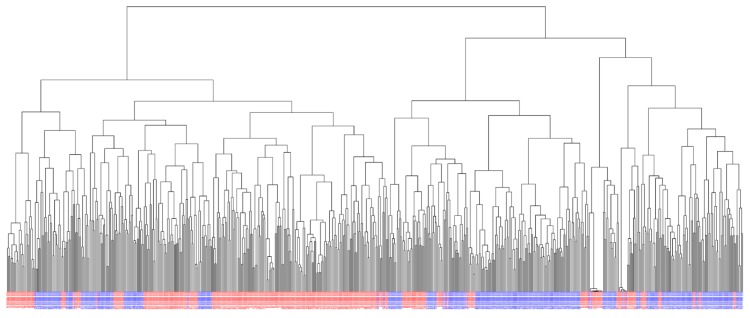
Dendrogram of the 635 wheat lines based on the G-matrix. Lines from set2014 are shown in red and lines from set2015 are shown in blue.

A total of 635 F_6_ winter wheat lines from two different breeding cycles (set2014 and set2015) were phenotyped for the quality traits flour yield and Alveos P, L, and W (Table S1). The phenotypic distribution for each trait is shown in Figure 1. Phenotypic variation was higher for the Alveograph traits than for flour yield (Table 1). The wheat lines were genotyped for 10,802 SNPs (Table S2). A G-matrix was computed using all SNPs. A dendrogram based on the G-matrix showed that the lines were genetically related both within and between sets (Figure 2). In total, the two sets consisted of 159 full-sib families with an average of four full-sibs per family.

For all traits, additive genetic variance was observed. Variance components were estimated based on GBLUP models and were used for estimation of narrow-sense genomic heritabilities. Heritabilities ranged from 0.38 for flour yield to 0.72 for Alveo W (Table 2).

### 3.2. GWAS

**Figure 3:**
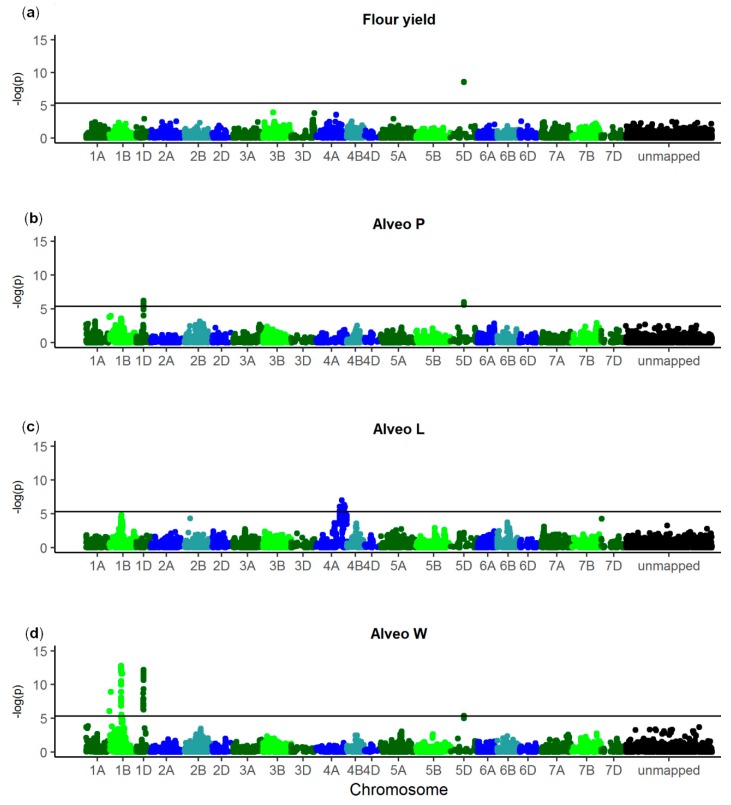
Manhattan plots of −log_10_(*p*-values) after correction with the genomic inflation factors. (**a**): Flour yield, (**b**): Alveo P, (**c**): Alveo L, (**d**): Alveo W. The horizontal lines show the Bonferroni corrected significance threshold. Total number of single nucleotide polymorphisms (SNPs): 10,802. Last bin is unmapped SNPs.

Single marker regression was performed for each of the 10,802 SNPs (Figure 3). Two linked SNPs on chromosome 5DS were significantly associated with flour yield, Alveo P, and Alveo W. On chromosome 1DL, significantly associated SNPs were identified for Alveos P and W. Additionally, SNPs associated with Alveo W were identified both on the short arm and on the long arm of chromosome 1B. A region on chromosome 4AL was significantly associated with Alveo L. The frequencies of the SNP alleles that were positively associated with each trait ranged from 13% to 64% (Table 3). A large difference was observed in Alveo P and in Alveo W for lines with the positive alleles of all significant SNPs compared to lines with the negative allele of one or more of the SNPs (Table 4 and Table 5).

**Figure 4:**
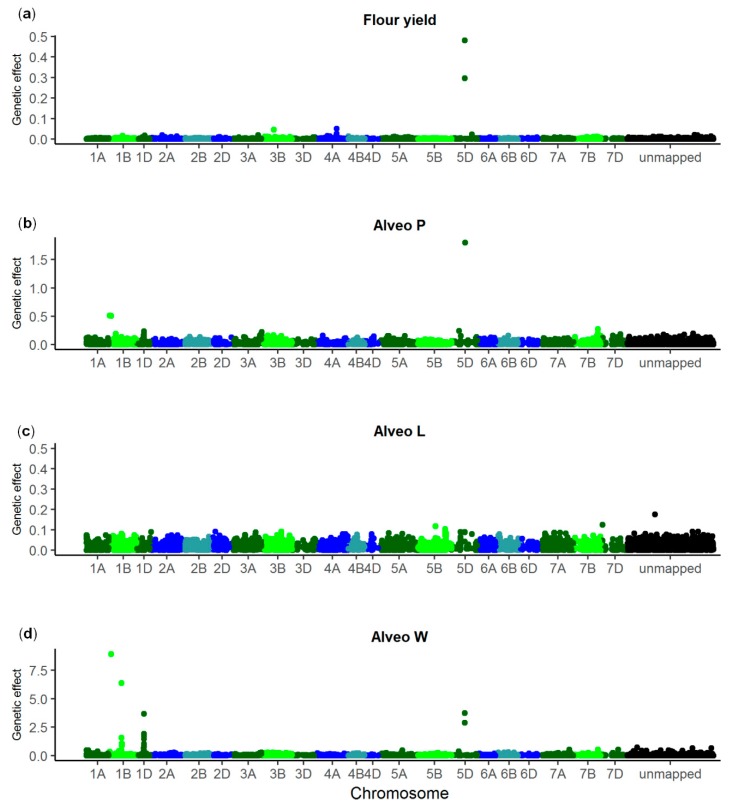
Genetic effect of SNPs estimated by the Bayesian Power Lasso models. (**a**): Flour yield, (**b**): Alveo P, (**c**): Alveo L, (**d**): Alveo W. Total number of SNPs: 10,802. Last bin is unmapped SNPs.

GWAS were also performed using Bayesian Power Lasso models to fit all 10,802 SNPs simultaneously [50]. The optimal value for *β* was 0.4 for flour yield and for Alveo W, 0.6 for Alveo P, and 0.8 for Alveo L, respectively. The SNPs most significantly associated with flour yield, Alveo P, and Alveo W according to the single marker regressions were also the SNPs with the highest genetic effects according to the Bayesian Power Lasso models (Figure 4). For Alveo L, each SNP had very low genetic effect (less than 0.2).

### 3.3. Genomic Predictions

**Figure 5:**
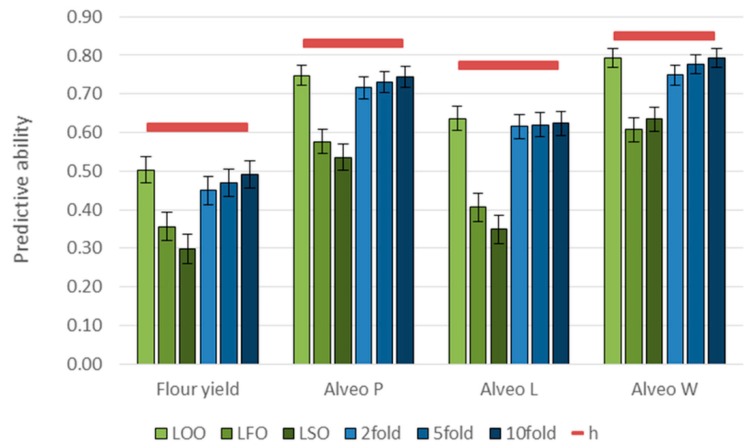
Predictive abilities for each quality trait based on GBLUP models. Red lines are the square root of narrow-sense genomic heritabilities (h) for each trait. Cross-validation (CV) strategies: LOO= leave-one-out, LFO= leave-family-out, LSO= leave-set-out, and k-fold= 2-, 5-, and 10-fold.

Genomic predictions based on all 10,802 SNPs using a GBLUP model were evaluated using different CV strategies. Predictive abilities of the models were determined as the correlations between observed phenotypes corrected for fixed effects and the GEBVs. The predictive abilities were intermediate to high, ranging from 0.50 for flour yield to 0.79 for Alveo W based on the LOO CV (Figure 5). The LFO and the LSO CVs resulted in lower predictive abilities. The lowest predictive ability was 0.3 for flour yield based on the LSO CV. The predictive abilities of the k-fold CVs increased slightly when using a higher number of folds, and they were very close to the LOO when using 10 folds. The predictions were unbiased for the LOO and the k-fold CV and only slightly biased for the LFO and the LSO (Table 6).

**Figure 6:**
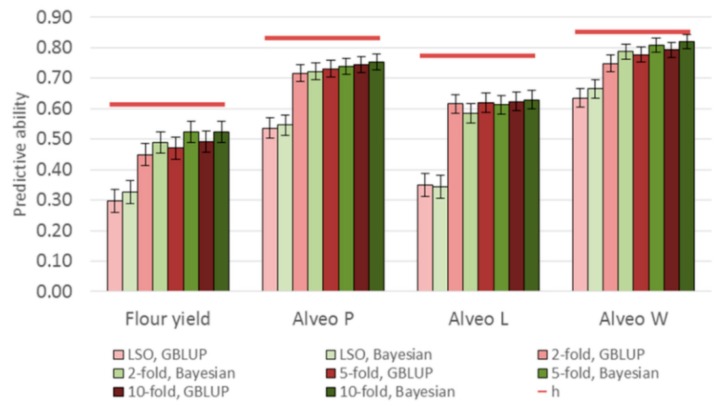
Comparison of predictive abilities based on GBLUP and Bayesian Power Lasso models. Red lines are the square root of narrow-sense genomic heritabilities (h) for each trait based on the GBLUP models.

For every CV strategy, the predictive abilities of the Bayesian Power Lasso model were a little better compared to the GBLUP for flour yield, Alveo P, and Alveo W, but not for Alveo L (Figure 6).

**Figure 7:**
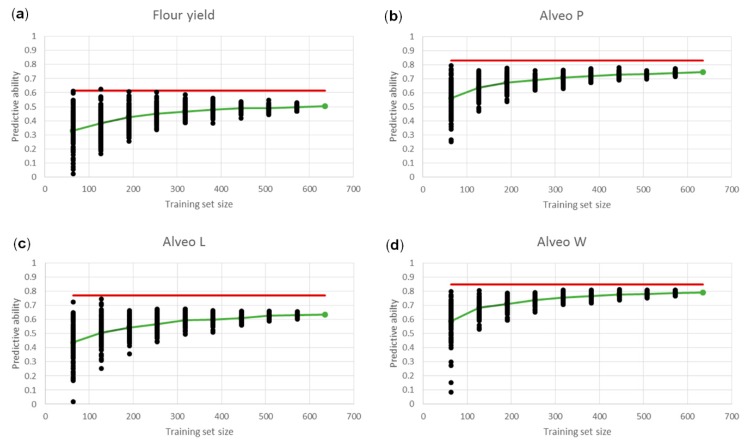
Predictive abilities based on leave-one-out cross-validations using reduced training set sizes. For each training set size, lines were selected randomly 100 times, and the average predictive abilities are shown with the green lines. The red lines are the square root of the narrow-sense genomic heritabilities based on all lines. (**a**): Flour yield, (**b**): Alveo P, (**c**): Alveo L, (**d**): Alveo W.

The effects of training population sizes ranging from 10% to 90% of the 635 lines were studied (Figure 7). Predictive abilities increased from 0.33 at 10% (64 lines) to 0.50 at 90% (572 lines) for flour yield. For all traits, the predictive abilities increased when increasing the size of the training set, but the increases were smaller at larger sizes. Additionally, the variation around the mean of the predictive abilities decreased when increasing the size of the training set.

**Figure 8:**
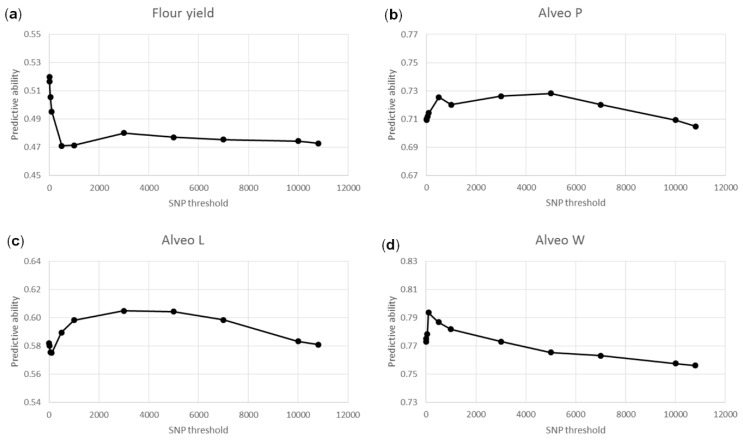
Predictive abilities based on genomic feature models with two G-matrices. The two G-matrices were computed from significant and nonsignificant SNPs, respectively. Half of the lines were used for GWAS to select significant SNPs, and the other half were used for LOO cross-validations. The threshold for number of significant SNPs was from five to 10,802. (**a**): Flour yield, (**b**): Alveo P, (**c**): Alveo L, (**d**): Alveo W.

Genomic features models with two G-matrices were also tested (Figure 8). One G-matrix was calculated from significant SNPs, and the other G-matrix was calculated from nonsignificant SNPs. The number of SNPs to include as significant ranged from five to all 10,802 SNPs. For flour yield, Alveo P, and Alveo W, predictive abilities were highest (0.52, 0.73, and 0.79, respectively) when fewer than 1000 SNPs were considered significant (10, 500, and 100 SNPs, respectively). For Alveo L, predictive abilities were highest (0.61) when 3000 SNPs were considered significant. For all traits, predictive abilities were higher when using the optimal number of significant SNPs for one G-matrix and the remaining SNPs for another G-matrix compared to using all SNPs for one G-matrix: 0.52 vs. 0.47 for flour yield, 0.73 vs. 0.70 for Alveo P, 0.61 vs. 0.58 for Alveo L, and 0.79 vs. 0.76 for Alveo W.

## 4. Discussion

Wheat quality traits typically have intermediate or high heritabilities, although the traits can be considerably affected by environmental effects and genotype–environment (G×E) interactions [36,37,38]. Thus, additive genetic variation across environments also affects the traits. Here, narrow-sense genomic heritabilities ranged from 0.38 for flour yield to 0.72 for Alveo W (Table 2). Therefore, breeding for improved wheat quality traits should be possible.

Advanced breeding material was used in the present study. The breeding program has, until now, focused more on increasing yield rather than improving baking quality due to restrictions in application of nitrogen fertilization to the fields in Denmark, which have made it challenging to grow high quality bread wheat lines. Nevertheless, variation was observed for the quality traits, indicating that both high and low quality wheat lines were used as crossing parents for the studied lines, and that genetic variation was maintained throughout the breeding program (Figure 1, Table 2). Six of the 96 crossing parents were used in both breeding sets. The dendrogram of the wheat lines indicated genetic relationships within each of the two sets (Figure 2). However, the lines from each set were not clearly divided in the dendrogram, indicating that the lines were also related between the two breeding sets.

QTL for wheat quality traits were identified across the genome [1,2,17]. However, many QTL were only identified in certain environments or populations [55,56]. In the present study, two closely linked SNPs on chromosome 5D were significantly associated with flour yield, Alveo P, and Alveo W (Figure 3). The puroindoline genes *Pina-D1* and *Pinb-D1* have a large effect on grain hardness. These genes are located on chromosome 5D and can affect several quality traits [1,12,57]. Several *Pinb-D1* alleles with a positive effect on wheat quality were identified [58,59]. However, relatively few of the markers in the present study were located on chromosome 5D [37], thus additional markers or sequencing would be needed to distinguish between each of the alleles.

For Alveo P and Alveo W, significant SNPs were also identified on chromosome 1D. The HMWG loci *Glu-D1* is located on chromosome 1D and has a large influence on wheat baking quality [4]. Similarly, the glutenin loci *Glu-B1* and *Glu-B3*, which are located on chromosome 1B, can affect quality. No SNPs on chromosome 1A were significantly associated with the Alveograph traits. The loci *Glu-A1* and *Glu-A3* are also known to affect wheat quality traits [4]. Thus, these loci might not be polymorphic in the studied material, their effects might be too low to detect, or the genotyped SNPs might not be located in or close enough to the loci. Several markers may be required in order to distinguish between the alleles of each of the *Glu* loci [60]. Therefore, the SNP chip array used to genotype the studied wheat lines can perhaps not capture all the genetic variants. Characterization of *Glu*- and *Pin* loci in the breeding material could be useful for more accurate estimation of their effects and consequently more accurate predictions of the baking quality traits [4,38,58].

The GWAS indicated that only few QTL with large or intermediate effects controlled the quality traits. The identified QTL only explained a relatively small proportion of the total genetic variance. This indicates that the traits were also controlled by many QTL with small effects. Identification of such minor QTL is challenging, especially if they have a low MAF or if they are located near major QTL. Lines with the positive alleles of the four largest QTL for Alveo W had considerably higher dough strength than lines with negative alleles of any of the QTL (Table 5). However, the four QTL together explained only 26.3% of the additive genetic variance based on the effects estimated from the single marker regressions, and these effects were possibly overestimated due to the Beavis effect [61,62]. The effects were lower when estimated using the Bayesian Power Lasso (Figure 4). Here, all SNP effects were estimated simultaneously, thus the effects were shrunk towards zero, and each QTL effect might have been distributed across several SNPs.

Since only a relatively small proportion of the genetic variance could be explained by the identified QTL, genomic predictions based on a large number of genome-wide markers could be useful. The different CV strategies showed that the predictive abilities were affected by size of the training set, by the genetic relationship between lines in training and validation sets, and by the G×E interactions (Figure 5). The LSO strategy represents one way of implementing genomic predictions in breeding programs. Predicting GEBVs of new lines based on lines from previous years could possibly enable selection before any phenotypic information is available for the new lines. However, the LSO strategy resulted in lower and more biased predictive abilities than the other CV strategies (Figure 5, Table 6). Possible reasons for the lower predictive abilities could be the size of the training set, the genetic relationship between lines, and the G×E interactions. Including lines from the same year and the same families in the training set improved the predictive abilities considerably. Reducing the size of training sets had a negative impact on the predictive abilities (Figure 7). However, the decrease for very small training sets seen in the present study was not as drastic as in other studies [29,32], possibly due to the high heritabilities of the traits included in this study. Thus, a few hundred lines might be enough for the training set, if they are highly related to the validation set. The G×E interactions and the genetic relationship between lines might be partly confounded, since the lines were only tested from one location. These effects had a larger impact on predictive abilities than the size of the training set and the model used for the predictions. G×E interactions can be accounted for in, for example, reaction norm models if phenotypic data are available for lines replicated across several locations or years. Additionally, data about climatic conditions and soil types might be included to obtain higher predictive abilities [63]. Such models could also be used for selecting lines for target environments or lines that are performing well over many environments. Predictive abilities can also be affected by the number of markers used for the predictions [29,32]. If the markers are selected based on GWAS, the number can possibly be reduced to a few hundred markers without affecting the predictive abilities. However, the markers that are selected would not be the same for each trait, and the markers could change after each new breeding cycle [29].

The predictive abilities of the Bayesian Power Lasso models were slightly higher compared to the GBLUP models for flour yield, Alveo P, and Alveo W (Figure 6). In the Bayesian Power Lasso model, the difference between small and large QTL effects can be bigger than in GBLUP [50]. Thus, the largest QTL effects might be shrunk too much in the GBLUP models. For Alveo L, no improvements were observed when using the Bayesian models, indicating that no major QTL were present for this trait (Figure 6). Previous studies have also shown that different types of Bayesian models can, in some cases, give slightly more accurate predictions compared to GBLUP models, especially for traits influenced by major QTL and for populations with low genetic relationships between training and validation sets [26,37,50].

Other studies of genomic selection for wheat quality traits using breeding material have reported predictive abilities in similar ranges as in the present study [36,37,64]. Thus, genomic prediction appears to be a promising strategy for improving wheat quality in breeding programs. Nevertheless, the use of information from GWAS or from known major QTL in genomic predictions might be useful for modeling marker effects more accurately. Markers for major QTL can be specified as having fixed effects, while remaining markers have random effects. Bernardo (2014) [39] recommended to specify fixed effects for markers that explain more than 10% genetic variance for traits controlled by fewer than 10 major genes. Zhao et al. (2014) [26] used W-BLUP (weighted BLUP) to give few functional markers a larger weight than other markers. This improved prediction accuracies of heading time and plant height in hybrid wheat compared to marker-assisted selection (MAS), ridge regression BLUP (equivalent to GBLUP) and BayesCπ. Improved prediction accuracies were also reported by Arruda et al. (2016) [34] when using fixed effects for QTL associated with Fusarium head blight resistance traits compared to ridge regression BLUP, and similarly for pre-harvest sprouting tolerance by Moore et al. (2017) [40]. However, including QTL identified using the same lines that were also used for the genomic predictions could lead to inflation of the accuracies [34]. In a recent study by Michel et al. (2018) [38], prediction accuracies for several wheat quality traits were higher based on ridge regression BLUP compared to specifying fixed effects for associated markers identified in independent populations. However, including one or more of the three *Glu-1* loci as fixed effects resulted in improved accuracies. For each trait, *Glu-1* loci were included if the locus explained more than 5% genetic variance [38].

In the present study, an alternative approach using one G-matrix computed from the most significant SNPs and another G-matrix computed from the remaining SNPs could slightly improve predictive abilities (Figure 8). However, the optimal significance threshold depended on the trait. For the traits controlled by few QTL with large effects, a conservative significant threshold seemed to be best, while a loose threshold seemed to be better for traits controlled only by QTL with small effects. To avoid inflation of the predictive abilities, half of the lines were used for the GWAS to select significant SNPs, and the other half were used for the genomic predictions. Thus, larger datasets might be necessary to study the predictive abilities of the genomic feature models and the optimal number of SNPs more thoroughly.

Implementation of genetic markers and genomic predictions in breeding programs could likely lead to increased genetic gains for the wheat quality traits. Resources needed for phenotyping could be reduced, and the selection intensity could be increased. The identified SNPs with large effects might be used for screening large numbers of lines early in the breeding program and selecting lines that potentially have good quality traits. Genomic predictions could be used for a more accurate selection of lines with good quality in later generations.

## 5. Conclusions

SNPs significantly associated with flour yield, Alveo P, and Alveo W were identified on chromosome 5D. For Alveos P and W, associated SNPs were also identified on chromosome 1D. Likely candidate genes could be the *Pina-D1* or the *Pinb-D1* on chromosome 5D and the *Glu-D1* loci on chromosome 1D. Furthermore, SNPs associated with Alveo W were identified on chromosome 1B, and SNPs associated with Alveo L were identified on chromosome 4A. Additive genetic variance explained by a single SNP was up to 13.3% (SNP on 5D for flour yield). The identified SNPs can be used in early generations of breeding programs to screen large numbers of lines. In later generations, it would be advantageous to use a large number of SNPs to ensure accurate prediction of breeding values. Predictive abilities of GBLUP models were 0.50 for flour yield, 0.75 for Alveo P, 0.79 for Alveo W, and 0.64 for Alveo L based on the LOO CV. Predictive abilities were lower when using smaller training sets but were still moderate when using only 10% of the 635 lines as the training set. Furthermore, predictive abilities were significantly lower when using LSO and LFO CV strategies because of reduced genetic relationship between lines in training and validation sets and because of G×E interactions. Predictive abilities were similar or slightly higher based on Bayesian Power Lasso and genomic feature models. Thus, GBLUP models could be used for genomic prediction of wheat quality traits with moderate to high predictive ability. Other models might give slightly higher predictive abilities for traits where major QTL are present in the breeding material.

## Figures and Tables

**Table 1:** Mean, range, coefficient of variation of phenotypic data.

Trait	Mean	Range	Coefficient of Variation (%)
Flour yield (%)	69.9	60.8–77.3	4.3
Alveo P (mm)	73.8	31–201	32.5
Alveo L (mm)	56.1	21–114	28.0
Alveo W (10^−4^ J)	134.2	40–293	33.4

**Table 2:** Additive genetic variance components (Var_g_), residual variance components (Var_e_), and narrow-sense genomic heritabilities (h^2^) estimated from genomic best linear unbiased prediction (GBLUP) models.

Trait	Var_g_	Var_e_	h^2^
Flour yield	2.8 ± 0.30	4.6 ± 0.36	0.38 ± 0.046
Alveo P	327.6 ± 23.7	146.8 ± 15.3	0.69 ± 0.053
Alveo L	138.6 ± 11.3	94.1 ± 8.9	0.60 ± 0.054
Alveo W	1108.3 ± 74.4	429.0 ± 45.3	0.72 ± 0.050

**Table 3:** SNPs significantly associated with each of the quality traits based on single marker regressions.

Trait	SNP ^1^	Chromo-some	*p* Value	Allele Frequency (%) ^2^	Genetic Effect	Explained Genetic Variance (%)	Gene ID	Annotation
Flour yield	NOS_WW_ SNP_688	5D	2.44 × 10^−9^	28	0.96 ± 0.14	13.3	TraesCS5D02G004300	Nontranslating coding sequence
Alveo P	NOS_WW_ SNP_5054	1D	6.22 × 10^−7^	15	7.99 ± 1.29	5.0	TraesCS1D02G322300	Uncharacterized protein
	NOS_WW_ SNP_688	5D	1.02 × 10^−6^	28	6.01 ± 0.99	4.4	TraesCS5D02G004300	Nontranslating coding sequence
Alveo L	NOS_WW_ SNP_2731	4A	9.59 × 10^−8^	37	4.87 ± 0.82	8.0	TraesCS4A02G447000	Glycosyl-transferase
Alveo W	NOS_WW_ SNP_11809	1B	1.77 × 10^−13^	64	14.58 ± 1.66	8.8	TraesCS1B02G329600	Uncharacterized protein
	NOS_WW_ SNP_6663	1B	1.21 × 10^−9^	64	12.04 ± 1.67	6.0	TraesCS1B02G016400	Uncharacterized protein
	NOS_WW_ SNP_3056	1D	6.30 × 10^−13^	13	20.18 ± 2.35	8.3	TraesCS1D02G317100	Histone deacetylase
	NOS_WW_ SNP_688	5D	4.16 × 10^−6^	28	9.41 ± 1.74	3.2	TraesCS5D02G004300	Nontranslating coding sequence

^1^ Only the most significant SNP for each peak in the Manhattan plots is shown. ^2^ Allele frequencies are for the allele that is positively associated with the trait.

**Table 4:** Mean values of Alveo P for lines with each combination of alleles of the significant SNPs. Alleles that are positively associated with the trait are marked with green.

NOS_WW_ SNP_5054 (Chr. 1D)	NOS_WW_ SNP_688 (Chr. 5D)	Alveo P (mm)	Number of Lines
G	C	64.0	386
G	T	84.2	125
A	C	100.7	43
A	T	104.7	36

**Table 5:** Mean values of Alveo W for lines with each combination of alleles of the significant SNPs. Alleles that are positively associated with the trait are marked with green.

NOS_WW_ SNP_3056 (Chr. 1D)	NOS_WW_ SNP_11809 (Chr. 1B)	NOS_WW_ SNP_6663 (Chr. 1B)	NOS_WW_ SNP_688 (Chr. 5D)	Alveo W (10^−4^ J)	Number of Lines
C	G	G	C	88.9	75
C	G	G	T	83.8	11
C	G	A	C	127.4	86
C	A	G	T	132.9	26
C	A	A	C	137.0	144
C	A	A	T	164.7	74
T	G	G	C	131.8	5
T	G	G	T	127.0	3
T	G	A	C	160.2	5
T	G	A	T	167.3	3
T	A	G	C	145.5	6
T	A	G	T	170.5	2
T	A	A	C	204.7	14
T	A	A	T	214.5	26

**Table 6:** Regressions of corrected phenotypes on genomic estimated breeding values (GEBVs) based on different cross-validation strategies using the GBLUP models. Bias is the deviation from the expected regression of 1.0.

Trait	LOO ^1^	LFO	LSO	2-Fold	5-Fold	10-Fold
Flour yield	1.00 ± 0.07	0.89 ± 0.09	0.71 ± 0.09	0.95 ± 0.08	0.97 ± 0.07	0.99 ± 0.07
Alveo P	1.00 ± 0.04	0.98 ± 0.06	0.90 ± 0.06	1.02 ± 0.04	0.99 ± 0.04	1.00 ± 0.04
Alveo L	0.99 ± 0.05	0.94 ± 0.08	0.81 ± 0.09	1.03 ± 0.05	0.97 ± 0.05	0.98 ± 0.05
Alveo W	1.02 ± 0.03	0.95 ± 0.05	0.96 ± 0.05	1.03 ± 0.04	1.02 ± 0.03	1.03 ± 0.03

^1^ Cross-validation strategies: leave-one-out (LOO), leave-family-out (LFO), leave-set-out (LSO), and k-fold: 2-, 5-, and 10-fold.

## Supplementary Materials

The following are available online at https://www.mdpi.com/2073-4425/10/9/669/s1, Table S1: Phenotypic data, Table S2: Genotypic data.
